# High Protein Diets Improve Liver Fat and Insulin Sensitivity by Prandial but Not Fasting Glucagon Secretion in Type 2 Diabetes

**DOI:** 10.3389/fnut.2022.808346

**Published:** 2022-05-19

**Authors:** Jiudan Zhang, Olga Pivovarova-Ramich, Stefan Kabisch, Mariya Markova, Silke Hornemann, Stephanie Sucher, Sascha Rohn, Jürgen Machann, Andreas F. H. Pfeiffer

**Affiliations:** ^1^Department of Endocrinology, Diabetes and Nutrition, Charité – Universitätsmedizin Berlin, Berlin, Germany; ^2^Department of Clinical Nutrition, German Institute of Human Nutrition Potsdam-Rehbruecke (DIfE), Potsdam, Germany; ^3^Deutsches Zentrum für Diabetesforschung (DZD), Neuherberg, Germany; ^4^Hamburg School of Food Science, Institute of Food Chemistry, University of Hamburg, Hamburg, Germany; ^5^Faculty of Process Sciences, Institute of Food Technology and Food Chemistry, Technical University of Berlin, Berlin, Germany; ^6^Section on Experimental Radiology, Department of Diagnostic and Interventional Radiology, University Hospital, Tübingen, Germany; ^7^Institute for Diabetes Research and Metabolic Diseases (IDM) of the Helmholtz Center Munich at the University of Tübingen, Tübingen, Germany

**Keywords:** glucagon, insulin sensitivity, liver fat content, alanine, type 2 diabetes, non-alcoholic fatty liver disease (NAFLD), high protein diet

## Abstract

Glucagon (GCGN) plays a key role in glucose and amino acid (AA) metabolism by increasing hepatic glucose output. AA strongly stimulate GCGN secretion which regulates hepatic AA degradation by ureagenesis. Although increased fasting GCGN levels cause hyperglycemia GCGN has beneficial actions by stimulating hepatic lipolysis and improving insulin sensitivity through alanine induced activation of AMPK. Indeed, stimulating prandial GCGN secretion by isocaloric high protein diets (HPDs) strongly reduces intrahepatic lipids (IHLs) and improves glucose metabolism in type 2 diabetes mellitus (T2DM). Therefore, the role of GCGN and circulating AAs in metabolic improvements in 31 patients with T2DM consuming HPD was investigated. Six weeks HPD strongly coordinated GCGN and AA levels with IHL and insulin sensitivity as shown by significant correlations compared to baseline. Reduction of IHL during the intervention by 42% significantly improved insulin sensitivity [homeostatic model assessment for insulin resistance (HOMA-IR) or hyperinsulinemic euglycemic clamps] but not fasting GCGN or AA levels. By contrast, GCGN secretion in mixed meal tolerance tests (MMTTs) decreased depending on IHL reduction together with a selective reduction of GCGN-regulated alanine levels indicating greater GCGN sensitivity. HPD aligned glucose metabolism with GCGN actions. Meal stimulated, but not fasting GCGN, was related to reduced liver fat and improved insulin sensitivity. This supports the concept of GCGN-induced hepatic lipolysis and alanine- and ureagenesis-induced activation of AMPK by HPD.

## Introduction

Glucagon (GCGN) increases glucose production in the liver, stimulates insulin release from beta cells and contributes to maintaining normal levels of glucose in a close interplay with insulin in healthy subjects (1, 2). Hyperglucagonemia was proposed as an early driver of hyperglycemia and as an initial step in the pathogenesis of type 2 diabetes mellitus (T2DM) (3, 4) although the causes of hyperglucagonemia remain controversial (5). Insulin resistance of the alpha-cell was proposed to impair the inhibition of glucagon secretion by insulin and may thereby increase GCGN levels (3). Glucagon release is directly and acutely stimulated by amino acids (AA) (6) and drives their hepatic degradation in the urea cycle (7, 8), which generates a liver-alpha-cell feedback loop. Non-alcoholic fatty liver disease (NAFLD) is a frequent consequence of obesity and associated with increased levels of AA (9) which was proposed to result from fatty liver-induced hepatic resistance to the GCGN-induced degradation of AA. The ensuing hyper-aminoacidemia may in turn stimulate GCGN-release and induce fasting and postprandial hyperglucagonemia in obesity and diabetes mellitus. The increase of fasting GCGN is thought to increase glucose production and to induce hyperinsulinemia which will further aggravate NAFLD and insulin resistance (10). The product of GCGN and alanine was recently proposed as an indicator of hepatic GCGN resistance and was associated with hepatic fat content (11). Fatty liver is closely linked to insulin resistance and increased levels of AAs, such that the overlap and interdependence of both phenomena make it difficult to separate the causes.

Although GCGN antagonists reduced blood glucose levels in T2DM patients they increased hepatic transaminases, induced fatty liver and dyslipidemia (5, 12–14). This raised awareness of the positive actions of GCGN such as the induction of lipolysis and lipid oxidation, inhibition of appetite and increase in energy expenditure (5, 15, 16). Moreover, recent work unraveled an important role of intra-islet GCGN release from alpha cells in maintaining beta cell responses (5, 17, 18). This work was backed by the development of GCGN agonists in peptide polyagonists combining GCGN, GLP-1, and/or GIP to treat T2DM (5). As AAs are potent inducers of GCGN secretion, high protein diets (HPDs) might be used to increase GCGN release and thereby profit from its benefits (16). Indeed, we recently tested HPDs without restriction of calorie intake in patients with T2DM and observed improvements of insulin sensitivity, hepatic fat content, circulating fatty acids, uric acid, and markers of inflammation and redox metabolism (19–23).

This raises the question, whether (a) fatty liver is quantitatively linked to fasting glucagon secretion and hepatic GCGN resistance in T2DM as reflected by elevated fasting AA and the GCGN–alanine index and (b), whether a reduction of liver fat would improve the hepatic GCGN resistance in people with T2DM as might be expected if NAFLD is a primary cause of hyperglucagonemia. As NAFLD is also closely linked to insulin resistance, the reduction of liver fat should improve alpha-cell insulin sensitivity and may thereby reduce fasting and postprandial GCGN release. Because alpha-cell-GCGN-stimulated insulin secretion is largely mediated by GLP-1 receptors, GCGN-resistance might not alter the response to protein- and AA intake-induced insulin secretion in mixed meal tolerance tests (MMTTs).

A second aspect arises from potential beneficial effects of GCGN in obesity and T2DM: GCGN specifically drives intrahepatic lipolysis and lipid oxidation through a recently discovered inositol trisphosphate-receptor-1 (INSP3-R1) dependent signal pathway and thereby is a powerful stimulus to reduce liver fat (24). Preclinical studies moreover suggest a centrally mediated inhibition of hepatic lipogenesis by GCGN (16). Indeed, isocaloric HPDs which strongly stimulate GCGN release, have been used to reduce liver fat in patients with T2DM by over 40% which most likely was mediated by the increase in GCGN-induced hepatic lipolysis (19, 20). This raises the question whether GCGN resistance of the liver would impair the action of GCGN and thereby serve as a marker of the prospective effectiveness of HPD for the reduction of liver fat in people with NASH/NAFLD.

This analysis was performed to assess the interplay of intrahepatic lipids (IHLs) with plasma levels of GCGN and hepatic GCGN-resistance in study participants with T2DM before and after extensive loss of liver fat achieved by the intake of HPDs (30%E of protein) for 6 weeks. We assessed whether there is (a) a correlation of IHL with insulin sensitivity and GCGN resistance determined by the GCGN–alanine index at baseline and after the intervention, (b) whether an extensive reduction of IHL by isocaloric HPD affects insulin or GCGN sensitivity, (c) whether GCGN sensitivity at baseline determines the effect of the HPD on loss of IHL, and whether (d) GCGN sensitivity affects the secretion of insulin induced by a mixed meal, i.e., whether the ultra-short loop feedback between alpha- and beta-cells changes.

## Materials and Methods

The analysis is based on the “LeguAN” intervention trial in subjects (18–80 years) with T2DM, which was registered at ClinicalTrials.gov (NCT02402985). Participants with orally treated T2DM, matched for age, sex, body mass index (BMI), glycated hemoglobin A1c (HbA1c), and anti-diabetic medications, were randomized using computer algorithm to 6 weeks of isocaloric diets which contained 30% of energy intake (%E) as protein, 40%E as carbohydrates, and 30%E as fat (20). All participants received individually adapted dietary instructions and meal plans by an experienced dietician and Master in Nutrition (SS) and were partially supplied with foods during the 6 weeks. The overall composition of SAFA (10%E), MUFA (10%E), and PUFA (10%E) was kept similar as much as possible and dietary intake was calculated with the computer program PRODI as described in detail in the supplements of refs (19, 20). The study participants completed MMTTs before and at the end of the study which consisted of breakfast (MMTT1) and lunch (MMTT2) with detailed profiles of insulin, GCGN, glucose, and AA over 360 min. The original study compared plant vs. animal protein rich diets which showed similar improvements of IHL, insulin sensitivity, fasting glucose, HbA1c, visceral adipose tissue (VAT), inflammatory, liver, and redox markers ref (19–23). The groups were therefore combined in the current analysis. The separation into two groups with changes of liver fat above vs. below the median comprised animal/plant protein of 7/8 in the higher and 9/7 in the lower liver fat change groups. Changes of protein intakes, blood urea nitrogen (BUN) and urinary nitrogen excretion relative to changes in IHL, GCGN, and homeostatic model assessment for insulin resistance (HOMA-IR) are shown in Supplementary Figures 2–4. The free fatty acid (FFA) in serum showed a decrease of all saturated fatty acids (C14–C22), no change of linoleic acid and a small increase of alpha-linoleic acid as reported previously (20). All subjects signed informed consent prior to participation. A total of 31 subjects were included who performed proton magnetic resonance spectroscopy (^1^H-MRS) of the liver and MRI for VAT on a 1.5 T whole body imager (Magnetom Avanto, Siemens Healthcare, Erlangen, Germany) at baseline and after 6-weeks of high-protein dietary intervention (19–21). Body composition (fat mass and lean mass) was determined by Air Displacement Plethysmography (BOD POD, COSMED, Italy). Routine parameters were measured in serum using ABX Pentra 400 (Horiba, Japan). Insulin and glucagon in serum samples were measures by ELISA (Mercodia, Sweden). Plasma AA levels were determined by liquid chromatography tandem mass spectrometry analysis.

### Calculations

Index of whole-body insulin resistance (HOMA-IR) was calculated as: fasting insulin (mU/L) × fasting glucose in (mmol/L)/22.5 (25). Matsuda index was calculated according to Matsuda and DeFronzo (26).

The GCGN–alanine index and the GCGN–AA-index were calculated as fasting glucagon × fasting alanine or other AA, respectively, according to the previous publication (12). The glucose disposal rate (*M*-value) was calculated from the infusion rate of exogenous glucose during steady state of the hyperinsulinemic euglycemic clamp (HEC) as previously described.

### Statistical Analysis

For statistical analysis, all variables are described as mean ± SD. Normal distribution was evaluated by Shapiro–Wilk-test. According to the normal or non-normal distribution, statistical comparison of variables at baseline and after 6-weeks high protein intervention between two groups was performed by independent *t*-test or Mann–Whitney *U*-test; Paired *t*-test or Wilcoxon signed rank test was used within groups. The repeated measures ANOVA was used to analyze differences at different time-points.

For correlation analysis, non-normally distributed data (GCGN–AA index, IHL, and HOMA-IR) were logarithmically transformed to approximate a linear distribution. Spearman’s non-parametric rank or Pearson correlations were conducted depending on the normality of data distribution. Areas under the curve (AUC) and incremental areas under the curve (iAUC) were calculated by GraphPad prism 8 (CA, United States) using the trapezoid rule.

A *p*-value < 0.05 was considered statistically significant. All statistical calculations were performed using SPSS 26.0 (IBM, United States). All graphs were generated by GraphPad prism 8 (CA, United States).

## Results

**FIGURE 1:**
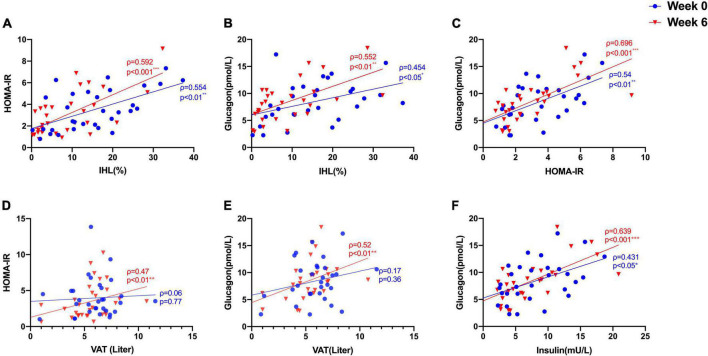
Correlations between **(A)** IHL (%) and insulin sensitivity (HOMA-IR); **(B)** IHL (%) and GCGN; **(C)** HOMA-IR and GCGN; **(D)** visceral adipose tissue (VAT) and HOMA-IR; **(E)** VAT and GCGN; **(F)** correlations between fasting insulin and GCGN before (Week 0, blue) and after high protein intake intervention in the entire study group (Week 6, red). *p < 0.05; **p < 0.01; ***p < 0.001.

We studied 31 study participants with orally treated T2DM whose characteristics are shown in Table 1. The intrahepatic lipid content (IHL) was 15.4 ± 9.8% determined by ^1^H-MRS and correlated highly with insulin sensitivity measured as HOMA-IR (ρ = 0.554, *p* = 0.001) (Figure 1A) and with fasting GCGN levels (ρ = 0.454, *p* = 0.012) (Figure 1B). VAT, determined by MRI, did not correlate with GCGN (ρ = 0.17, *p* = 0.36) (Figure 1E). The intervention resulted in markedly reduced liver fat content by 6.6%, slightly but significantly reduced VAT and significant improvements of HbA1c, fasting glucose, and insulin sensitivity (HOMA-IR, Matsuda index, and *M*-value) (Table 1) (19, 20). The levels of fasting GCGN did not change significantly (Table 1).

**TABLE 1:** Parameters at baseline (Week 0) and after the HPD intervention of all study participants (Week 6).

Parameter (*n* = 31)	Week 0	Week 6	*p*-Value
Age (years)	64.6 ± 6.0		
Gender (male/female)	19 m/12 f		
Liver fat content (MR-S; %)	15.4 ± 9.8	8.8 ± 8.1	<0.001***
Body weight (kg)	89.4 ± 14.2	87.4 ± 14.0	<0.001***
BMI (kg/m^2^)	30.6 ± 3.7	29.9 ± 3.5	<0.001***
Waist circumference (cm)	102.9 ± 10.9	100.6 ± 10.7	<0.01**
Fasting glucose (mmol/L)	9.6 ± 1.5	8.8 ± 1.5	<0.001***
Fasting insulin (mU/L)	8.4 ± 4.7	7.9 ± 5.4	0.16
Fasting glucagon (pmol/L)	8.2 ± 3.5	8.4 ± 3.7	0.63
Fasting C-P (μg/L)	1.9 ± 0.8	1.9 ± 0.9	0.40
Insulin/glucagon ratio	1.1 ± 0.72	0.89 ± 0.42	0.056
C-P/glucagon ratio	0.27 ± 0.17	0.23 ± 0.08	0.23
iAUC glucagon (pmol/L)	992.1 ± 577.4	829.3 ± 502.3	0.313
HbA1c	6.8 ± 0.70	6.4 ± 0.69	<0.001***
HOMA-IR	3.5 ± 1.9	3.1 ± 2.0	<0.05*
Matsuda index	4.5 ± 3.1	5.0 ± 2.9	<0.05*
*M*-value	4.9 ± 2.1	5.5 ± 1.9	<0.01**
AST (U/L)	25.2 ± 8.7	21.8 ± 6.1	<0.01**
ALT (U/L)	28.2 ± 9.9	26.5 ± 8.4	0.13
AST/ALT ratio	0.87 ± 0.21	0.84 ± 0.19	0.54
GGT (U/L)	44.1 ± 26.2	30.8 ± 15.9	<0.001***
TG (mmol/L)	1.7 ± 0.59	1.6 ± 0.66	0.22
TC (mmol/L)	5.3 ± 0.97	4.62 ± 0.95	<0.01**
LDL-C (mmol/L)	3.4 ± 0.89	2.9 ± 0.85	<0.01**
HDL-C (mmol/L)	1.1 ± 0.26	0.96 ± 0.17	<0.01**
CREA (μmol/L)	81.3 ± 16.2	77.5 ± 16.7	<0.05*
BUN (mmol/L)	6.0 ± 0.95	7.8 ± 1.8	<0.001***
eGFR (mL/min/1.73 m^2^)	78.6 ± 15.2	82.6 ± 15.2	<0.05*
Urine urea (mmol/24 h)	403.0 ± 134.2	564.0 ± 200.2	<0.001***
VAT (L)	6.0 ± 2.1	5.8 ± 1.9	<0.01**
Fat mass (%)	35.8 ± 7.3	33.9 ± 7.0	<0.05*
Lean mass (%)	64.0 ± 7.3	66.2 ± 7.0	<0.05*

BMI, body mass index; C-P, C-peptide; iAUC, incremental area under curve; HbA1c, glycated hemoglobin A1c; HOMA-IR, homeostatic model assessment for insulin resistance; AST, aspartate aminotransferase; ALT, alanine aminotransferase; GGT, gamma-glutamyl transferase; TG, triglycerides; TC, total cholesterol; CREA, creatinine; BUN, blood urea nitrogen; eGFR, estimated glomerular filtration rate; VAT, visceral adipose tissue. *p < 0.05; **p < 0.01; ***p < 0.001.

### Correlation of Glucagon, Glucagon–Alanine Index, and Insulin Sensitivity With Intrahepatic Lipid and Visceral Adipose Tissue

**FIGURE 2:**
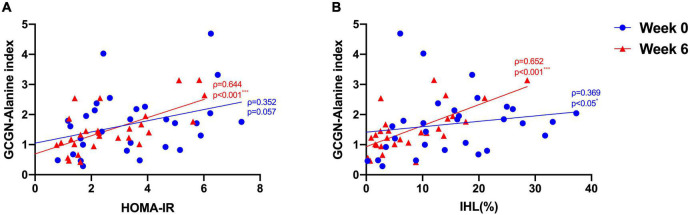
Correlations between **(A)** GCGN–alanine index and HOMA-IR; **(B)** GCGN–alanine index and IHL (%) before (Week 0, blue) and after the intervention in the entire study group (Week 6, red). *p < 0.05; ***p < 0.001.

Glucagon levels correlated with IHL and insulin sensitivity before and after the intervention (Figures 1B,C) and with VAT after the intervention (ρ = 0.52, *p* = 0.004) (Figure 1D). In order to assess hepatic GCGN sensitivity, we calculated the GCGN–alanine index as proposed (12) which correlated modestly with IHL at baseline (ρ = 0.369, *p* < 0.05). Insulin sensitivity calculated by HOMA-IR correlated trendwise and non-significantly with the GCGN–alanine index at baseline (ρ = 0.352, *p* = 0.057) (Figure 2A). Remarkably, the correlations of the GCGN–alanine index became highly significant upon the high protein intake for 6 weeks for IHL (ρ = 0.652, *p* < 0.001) (Figure 2B) and for insulin sensitivity (ρ = 0.644, *p* < 0.001) (Figure 2A). Similarly, increased correlations were observed between GCGN–alanine index and BCAA, glutamine, or histidine as well as between total AAs and with IHL or HOMA-IR (Supplementary Table 3). The intake of the high-protein diet thus greatly increased the alignment of GCGN and AA as reflected by their increasing correlation with liver fat and insulin sensitivity.

### Improvements of Insulin Sensitivity Upon Reduction of Liver Fat Are Dissociated From Changes of the Glucagon–Alanine Index

Glucagon is likely a key player in the protein-induced reduction of liver fat by high protein intake (24). The reductions of liver fat in our study showed large differences between individuals. We therefore hypothesized that these differences might be related to hepatic GCGN resistance resulting in impaired GCGN-induced hepatic lipolysis and induction of ureagenesis.

We therefore analyzed the participants according to changes above or below the median of liver fat change. This resulted in a significant difference of liver fat reduction between the groups although baseline levels of IHL did not differ significantly (Table 2). The lesser liver fat reduction group shifted from 17.4 to 12.7% IHL and thus maintained a high liver fat content even after the relative reduction by 27%. The greater liver fat reduction group decreased IHL by 65% from 13.3 to 4.6 ± 3.8% and thus – in average – below the defined threshold of fatty liver of 5.56% IHL. The modest reduction of weight and waist circumference was around 2 kg and 2 cm, respectively, identical in both groups as were modest reductions of visceral and total adipose tissue and modest increases in muscle mass (Table 2).

**TABLE 2:** Parameters at baseline (Week 0) and after HPD intervention (Week 6) of study participants with lower (below median) and higher (above median) reduction of intrahepatic lipid content (IHL).

Parameter	Lower liver fat reduction (*n* = 16) below median			Higher liver fat reduction (*n* = 15) above median			*p*week 6 vs week 0
	Week 0	Week 6	*p*	Week 0	Week 6	*p*	
Age (years)	63.0 ± 5.7			66.3 ± 6.0			
Gender (male/female)	8 m/8 f			11 m/4 f			
Liver fat content (MR-S; %)	17.4 ± 10.7	12.7 ± 9.2	<0.001***	13.3 ± 8.6	4.6 ± 3.8	<0.001***	<0.05*
Body weight (kg)	89.0 ± 14.0	86.7 ± 13.6	<0.001***	89.6 ± 15.6	86.8 ± 15.4	<0.001***	0.96
BMI (kg/m^2^)	31.0 ± 4.1	30.2 ± 4.0	<0.001***	30.2 ± 3.3	29.5 ± 3.1	<0.001***	0.96
Waist circumference (cm)	102.5 ± 10.4	100.7 ± 10.3	0.07	103.2 ± 11.8	100.6 ± 11.5	<0.01**	0.54
Fasting glucose (mmol/L)	9.3 ± 1.0	8.8 ± 1.1	<0.05*	10.0 ± 1.8	8.9 ± 1.8	<0.01**	0.12
Fasting insulin (mU/L)	8.4 ± 4.9	8.9 ± 6.4	0.28	8.3 ± 4.6	6.9 ± 4.1	<0.05*	<0.05*
Fasting glucagon (pmol/L)	8.2 ± 3.2	9.2 ± 4.0	0.24	8.7 ± 4.5	7.6 ± 3.4	0.51	0.18
Fasting C-P (ug/L)	1.9 ± 0.9	1.9 ± 1.0	0.59	1.8 ± 0.8	1.7 ± 0.8	0.07	0.11
AUC insulin (MMT1)	8915.3 ± 6880.0	9039.2 ± 7201.4	0.75	10163.1 ± 6425.7	8503.3 ± 4852.5	<0.05*	<0.05*
AUC insulin (MMT2)	6322.0 ± 4262.6	5923.3 ± 3508.7	0.14	6062.9 ± 4109.5	4926.7 ± 2844.5	0.06	0.35
AUC glucagon (MMT1)	2917.4 ± 869.5	3051.3 ± 1018.9	0.35	2925.1 ± 1004.8	2672.3 ± 1046.9	<0.05*	0.08
AUC glucagon (MMT2)	2988.1 ± 829.9	2755.9 ± 831.8	0.08	2651.7 ± 1089.3	2439.4 ± 1181.6	0.08	0.98
HbA1c	6.7 ± 0.54	6.3 ± 0.47	<0.01**	7.0 ± 0.81	6.6 ± 0.84	<0.05*	0.80
HOMA-IR	3.4 ± 1.9	3.4 ± 2.4	0.77	3.6 ± 2.0	2.6 ± 1.5	<0.01**	<0.05*
Matsuda index	4.8 ± 3.7	4.7 ± 2.9	0.72	4.2 ± 2.5	5.4 ± 3.0	<0.01**	<0.05*
*M*-value	5.0 ± 2.4	5.3 ± 2.0	0.28	4.8 ± 1.8	5.8 ± 1.7	<0.01**	0.11
AST (U/L)	26.4 ± 9.7	21.8 ± 5.8	<0.05*	24.0 ± 7.7	21.7 ± 6.5	0.16	0.34
ALT (U/L)	29.9 ± 12.7	27.8 ± 9.4	0.15	26.4 ± 5.5	25.1 ± 7.2	0.48	0.42
AST/ALT ratio	0.88 ± 0.24	0.82 ± 0.16	0.61	0.87 ± 0.18	0.86 ± 0.22	0.81	0.67
GGT (U/L)	48.4 ± 23.6	36.0 ± 17.9	<0.001***	39.5 ± 28.7	25.2 ± 11.5	<0.05*	0.81
TG (mmol/L)	1.7 ± 0.54	1.8 ± 0.74	0.33	1.7 ± 0.66	1.4 ± 0.52	<0.05*	<0.05*
TC (mmol/L)	5.2 ± 0.88	4.8 ± 1.0	<0.01**	5.4 ± 1.1	4.5 ± 0.88	<0.001***	<0.05*
LDL-c (mmol/L)	3.3 ± 0.86	3.0 ± 0.91	<0.05*	3.5 ± 0.94	2.9 ± 0.82	<0.01**	0.58
HDL-c (mmol/L)	1.1 ± 0.27	0.95 ± 0.14	<0.01**	1.2 ± 0.27	0.96 ± 0.21	<0.001***	0.18
Creatinine (μmol/L)	82.6 ± 17.5	79.9 ± 18.5	0.41	79.9 ± 15.2	74.9 ± 14.7	<0.05*	0.49
BUN (mmol/L)	6.0 ± 1.0	7.8 ± 1.7	<0.01**	5.9 ± 0.94	7.8 ± 1.9	<0.01**	0.38
eGFR (mL/min/1.73 m^2^)	77.1 ± 16.1	80.2 ± 15.9	0.38	80.3 ± 14.6	85.1 ± 14.6	<0.05*	0.61
Urine urea (mmol/day)	377.6 ± 79.3	507.6 ± 158.5	<0.01**	430.4 ± 175.0	624.7 ± 227.9	<0.01**	<0.05*
VAT (L)	6.0 ± 2.2	5.6 ± 2.1	<0.01**	5.9 ± 2.1	5.5 ± 1.9	0.12	0.92
Fat mass (%)	36.4 ± 9.0	34.8 ± 8.9	<0.01**	35.2 ± 4.9	32.6 ± 3.8	0.11	0.52
Lean mass (%)	63.6 ± 9.0	65.2 ± 8.9	<0.01**	64.8 ± 4.9	67.4 ± 3.8	0.11	0.52

BMI, body mass index; C-P, C-peptide; HbA1c, glycated hemoglobin A1c; HOMA-IR, homeostatic model assessment for insulin resistance; AST, aspartate aminotransferase; ALT, alanine aminotransferase; GGT, gamma-glutamyl transferase; TG, triglycerides; TC, total cholesterol; CREA, creatinine; BUN, blood urea nitrogen; eGFR, estimated glomerular filtration rate. *p < 0.05; **p < 0.01; ***p < 0.001.

Fasting glucose decreased significantly in both groups while fasting insulin decreased significantly in the greater liver fat reduction group only. Fasting GCGN did not change significantly in either group. Insulin sensitivity expressed by HOMA-IR, Matsuda index, or *M*-value improved significantly in the group with greater IHL reduction but not in the lesser IHL-reduction group resulting in a significant difference between the groups.

**FIGURE 3:**
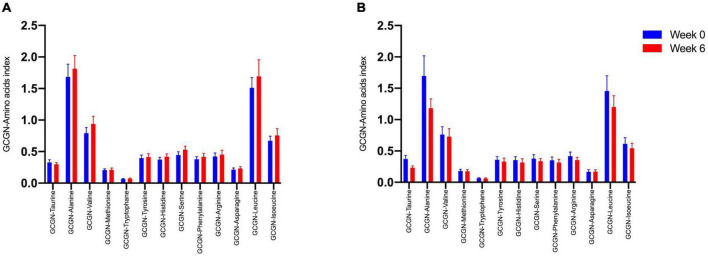
Glucagon–alanine index between **(A)** lower IHL reduction groups; **(B)** higher IHL reduction group before (Week 0, blue) and after the intervention (Week 6, red).

By contrast, the GCGN resistance indices calculated for alanine or AA did neither change significantly within, nor differ between the groups (Supplementary Tables 1, 2, Figure 3). However, the reduction of liver fat showed a borderline correlation with the change of GCGN (ρ = 0.344, *p* = 0.077) but not with the change of the GCGN–alanine index.

Notably, the correlations of the GCGN–AA indices with IHL and insulin sensitivity became highly significant for virtually all AA from baseline to follow-up, indicating a close alignment of GCGN-regulated AA-metabolism with IHL and insulin sensitivity (Supplementary Table 2). Thus, the reduction of liver fat is linked to a reduction of insulin resistance but not of GCGN resistance estimated by the GCGN–alanine index even upon extensive reductions of liver fat. However, the role of the GCGN–AA-hepatic axis appears to become enhanced which we interpret to reflect beneficial actions of GCGN.

### Does Glucagon Resistance Impair the High Protein Diet-Induced Loss of Liver Fat?

We then asked whether hepatic GCGN resistance may relate to impaired degradation of IHL by GCGN in response to HPD and therefore compared participants above with those below the median of the GCGN–alanine index regarding responses of IHL to high-protein diet. Indeed, the GCGN–alanine index in the upper half was associated with higher liver fat compared to the lower half both at baseline (20.9 ± 9.2 vs. 11.9 ± 9.4%; *p* < 0.05) and after 6 weeks (11.4 ± 7.3 vs. 4.1 ± 3.9%; *p* < 0.01). However, the absolute magnitude of liver fat reduction did not differ between the groups (6.8 ± 5.3 vs. 6.6 ± 5.1%; *p* > 0.05) and we did not find an indication that a higher GCGN–alanine index impairs the HPD-induced reduction of liver fat (Table 3 and Supplementary Figure 1).

**TABLE 3:** Parameters at baseline (Week 0) and after HPD intervention (Week 6) between lower (below median) and higher (above median) basal GCGN–alanine index groups.

	Week 0 Glucagon–alanine index			Week 6 Glucagon–alanine index		
Parameters	Lower (*n* = 16) below median	Higher (*n* = 15) above median	*p*	Lower (*n* = 16) below median	Higher (*n* = 15) above median	*p*
Liver fat content (MRS; %)	11.9 ± 9.4	20.9 ± 9.2	<0.05*	4.1 ± 3.9	11.4 ± 7.3	<0.01**
Insulin/glucagon ratio (fasting)	1.4 ± 0.84	0.85 ± 0.48	0.052	0.89 ± 0.42	0.83 ± 0.32	0.88
Insulin/glucagon ratio (60 min)	5.2 ± 3.7	2.9 ± 1.6	0.07	3.7 ± 2.0	4.2 ± 2.8	0.84
Insulin/glucagon ratio (120 min)	4.6 ± 2.8	3.5 ± 2.2	0.18	3.8 ± 2.0	4.3 ± 2.7	0.77
Insulin/glucagon ratio (180 min)	2.6 ± 1.6	2.4 ± 1.6	0.33	2.3 ± 1.1	2.4 ± 1.3	0.95

*p < 0.05; **p < 0.01; ***p < 0.001.

### Does Glucagon Play a Role for Circulating Free Fatty Acids?

We previously reported that HPDs reduced circulating saturated FFAs which associated with the changes in IHL (19). In view of the regulation of hepatic lipid metabolism by GCGN we assessed associations between circulating GCGN and FFA. Indeed, GCGN correlated significantly with palmitic and stearic acid and the *de novo* lipogenesis index, both before and after the intervention supporting a role of GCGN in the regulation of lipogenesis. This would be expected due to the AMPK induced inhibition of ACC (Table 4 and Supplementary Table 7). In agreement, there was no correlation with odd numbered FFA or unsaturated FFA or indices of desaturase or elongase activities (Supplementary Table 7).

**TABLE 4:** Correlations between GCGN and FFA and DNL-index before (Week 0) and after (Week 6) HPD intervention.

Parameters (*n* = 31)	Week 0	Week 6
C14:0	ρ = 0.253 *p* = 0.186	ρ = 0.417 *p* < 0.05*
C15.0	ρ = −0.071 *p* = 0.713	ρ = 0.094 *p* = 0.626
C17.0	ρ = 0.315 *p* = 0.096	ρ = 0.272 *p* = 0.153
C16:0	ρ = 0.388 *p* < 0.05*	ρ = 0.524 *p* < 0.01**
C18.0	ρ = 0.522 *p* < 0.01**	ρ = 0.489 *p* < 0.01**
DNLindex = 16:0/18:2n6	ρ = 0.531 *p* < 0.01**	ρ = 0.456 *p* < 0.05*

C14:0: myristic acid; C16:0: palmitic acid; C18:0: stearic acid; C15:0: pentadecanoic acid; C17:0: heptadecanoic acid; DNLindex: de novo lipogenesis index. *p < 0.05; **p < 0.01.

### Assessment of Beta-Cell Stimulation by High Protein Diet – Does the Glucagon Response Play a Role?

Capozzi and coworkers recently proposed that GCGN-induced insulin secretion contributes to lowering of blood glucose concentrations particularly in mixed meals (17, 18). We wondered whether changes of AAs and GCGN responses to protein challenges occurred in response to the reductions of liver fat by HPD. Fasting levels of AA did not change in response to the intervention. Fasting levels of GCGN and insulin were highly correlated (ρ = 0.431, *p* < 0.05), and the correlation increased markedly after the intervention (ρ = 0.639, *p* < 0.001) (Figure 1F).

**FIGURE 4:**
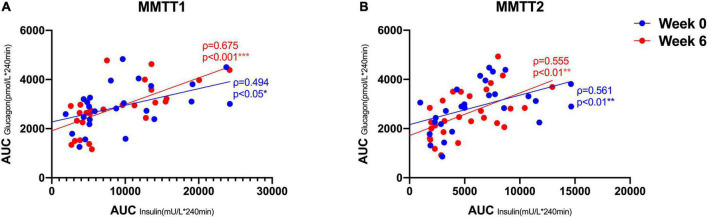
Correlations between AUC (*240 min) insulin and AUC (*240 min) GCGN in MMTT 1 **(A)** and MMTT 2 **(B)** before (Week 0, blue) and after the intervention (Week 6, red). *p < 0.05; **p < 0.01; ***p < 0.001.

**FIGURE 5:**
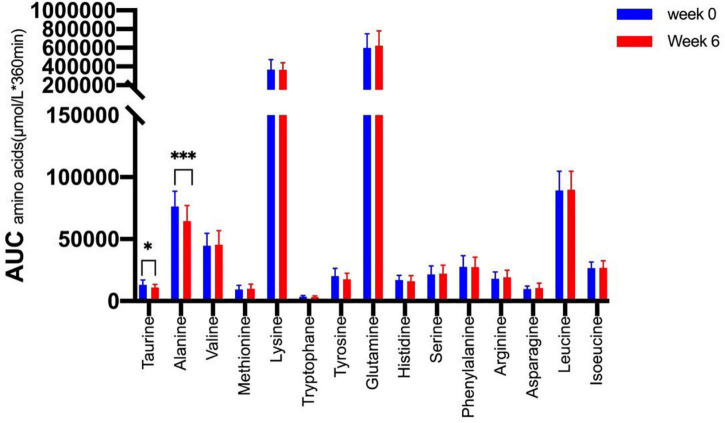
Area under the curve (*360 min) of amino acids in the MMTTs before (Week 0, blue) and after the intervention (Week 6, red). *p < 0.05; ^***^p < 0.001.

**FIGURE 6:**
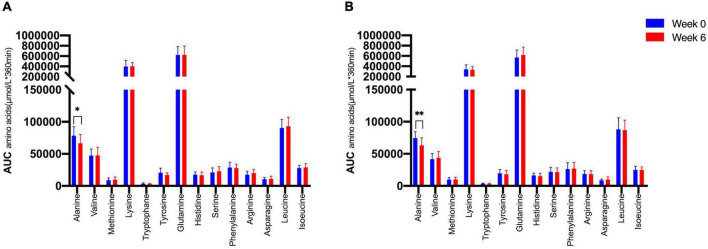
Area under the curve (*360 min) of amino acids in the MMTTs between **(A)** lower IHL reduction group; **(B)** higher IHL reduction group before (Week 0, blue) and after the intervention (Week 6, red). *p < 0.05; ^**^p < 0.01.

We therefore tested whether the AA and GCGN responses to intake of 30 g protein/mixed meal were related to the liver fat content by analyzing identical successive breakfast (MMTT1) and lunch (MMTT2) before and after the intervention. The reduction of IHL resulted in reduced overall responses of insulin and GCGN in the MMTTs (Table 5 and Figure 4). This was accompanied by significantly and selectively reduced increases of alanine but not of other AAs (Figure 5). We then performed the same calculations for the groups above and below the median with greater and lesser liver fat reduction. Indeed, the reductions of insulin-, GCGN-AUC in the meal tests were only observed in the greater liver fat reduction group while alanine-AUC was reduced in both groups (Table 2 and Figures 6A,B).

**TABLE 5:** Area under the curve-insulin and AUC-GCGN levels at baseline (Week 0) and after HPD intervention (Week 6) in MMTT 1 and MMTT 2.

Parameters	MMTT 1			MMTT2		
	Week 0	Week 6	*p*	Week 0	Week 6	*p*
AUC insulin	9482.6 ± 6410.0	8722.8 ± 5890.0	0.17	6233.7 ± 4015.9	5394.6 ± 3102.0	<0.05*
AUC glucagon	2917.4 ± 899.8	2856.6 ± 1007.4	0.51	2907.4 ± 964.2	2656.0 ± 979.8	<0.05*

AUC, area under the curve. *p < 0.05; **p < 0.01.

The insulin/GCGN ratios were, moreover, significantly higher in participants with a fasting and postprandial GCGN–alanine index below compared to above the median at baseline indicating relatively less GCGN release (Table 3). Remarkably, the insulin/GCGN ratio decreased markedly from 1.42 ± 0.84 to 0.89 ± 0.42 at 0 min and also over the meal test in the lower GCGN–alanine index group (with lower liver fat) indicating a greater relative secretion of GCGN. By contrast, the higher GCGN–alanine index group showed no significant change.

The ratios did not differ significantly between the higher and lesser IHL-reduction group before or after the intervention although the insulin/GCGN-ratio also decreased numerically in the greater liver fat reduction group (Supplementary Table 4).

## Discussion

The role of GCGN in the dysregulation of glucose and lipid metabolism is debated at present (5, 10, 15). GCGN is consistently elevated in people with fatty liver and T2DM and contributes to hyperglycemia as shown with GCGN antagonists (27, 28). However, GCGN antagonists increased dyslipidemia, IHL and liver enzymes (13). Glucagon selectively induces hepatic lipolysis and enhances insulin secretion within pancreatic islets (17, 24).

Our findings confirm (A) a positive correlation of fasting GCGN with hepatic fat content and insulin sensitivity in subjects with T2DM, obesity, and fatty liver. We report (B) that extensive, but not moderate, reductions of IHL after 6 weeks HPD induce the expected improvement in insulin sensitivity but do not alter fasting levels of GCGN. However, (C) postprandial stimulation of GCGN is reduced in parallel to reductions of insulin due to the better insulin sensitivity. However, the insulin/GCGN ratio in MMTTs decreased in participants with a greater reduction of liver fat and extensive metabolic improvements. Thus, the fasting and postprandial levels of GCGN relative to insulin increased indicating that higher GCGN responses were associated with metabolic improvements. Moreover, (D) a selective reduction of the postprandial response of alanine was observed which may indicate enhanced hepatic GCGN sensitivity which strongly regulates alanine metabolism (see below). Therefore, increasing GCGN by HPD indeed allows metabolic improvements which suggests that the beneficial actions of GCGN outweigh the negative role in glucose production and parallel the positive results of GCGN co-agonists.

### Correlation of Glucagon or the Glucagon–Alanine Index With Intrahepatic Lipid and Insulin Sensitivity

Glucagon is potently stimulated by increases of AA and then regulates not only the hepatic degradation of AA in the urea cycle but also increases hepatic glucose production and stimulates insulin secretion (5). This increase of glucose production is physiologically compensated for by increased insulin release and glucose disposal in healthy subjects (29) which remains functional in people with T2DM despite of an impaired insulin response to glucose (21).

The correlation of IHL with the GCGN–alanine index was borderline significant at baseline and GCGN alone showed a higher correlation with IHL than the GCGN–alanine index which thus reflects variable alanine levels. The correlation of the GCGN–alanine index with insulin sensitivity (HOMA-IR or *M*-value) was non-significant at baseline while a significant correlation with GCGN was observed. The GCGN–alanine index did not improve the association of GCGN with insulin sensitivity or fatty liver as might be expected if the dysregulation of fasting alanine plays a primary role. This was also true for all other GCGN–AA indices. As the GCGN–alanine index multiplies Ala (or other AA) with GCGN one would expect a higher correlation of the index than of GCGN alone if alanine contributes to the increased GCGN levels.

Remarkably, after the HPD intervention, the correlation of GCGN–alanine index with IHL and HOMA-IR became highly significant. This also applied to other GCGN–AA indices. We interpret this to reflect a greater impact of AA in the regulation of IHL and insulin sensitivity in combination with GCGN due to the increased protein intake. High protein intake results in the oxidation of AAs in muscle which employs the alanine–glucose (or Cahill) cycle to shuttle the amino groups to the liver for detoxification in the urea cycle (30). GCGN was shown to directly regulate both ALT enzymes (GPT and GPT-2) at the transcriptional level (31) as the first step of AA detoxification in the hepatic urea cycle. This may reflect the primary role of GCGN in the reduction of IHL due to GCGN induced hepatic lipolysis through the INSP3R1 mediated pathway which was recently described and provides an important explanation for the effect of HPD (24). The urea cycle was moreover shown to activate AMPK due to the consumption of ATP by arginino-succinate synthase which results in AMPK-induced inhibition of hepatic acetyl-CoA carboxylase and thus of lipogenesis (32). The high correlations of GCGN with saturated FFA and the *de novo* lipogenesis index support the role of GCGN in the regulation of lipid metabolism which became apparent in human studies with GCGN antagonist-induced dyslipidemia and fatty liver. Obviously, this also applies to HPD-induced increases of GCGN. The reduction of the metabolically toxic saturated FFA, in particular palmitic acid (C16:0) by HPD likely involves a regulation of *de novo* lipogenesis by GCGN, possibly due to inhibition of ACC1 by increased AMPK activity in the liver.

### Dissociation of Improvements of Fasting Insulin- and Glucagon-Sensitivity in Response to Reduced Intrahepatic Lipid

The associations of GCGN with increased fasting AA, insulin resistance and fatty liver appear to support its negative role in the obesity and diabetes-associated metabolic dysregulation. A stimulation of GCGN by high protein intake should therefore further deteriorate metabolism (10). The alternative view interprets the increase of GCGN as a defensive response in an attempt to reset metabolism (5). Indeed, the HPD induced marked improvements of metabolism (19, 20, 22). However, an extensive reduction of IHL by 42% upon consumption of HPD for 6 weeks did not alter fasting GCGN, AA-levels, or the GCGN–alanine index, indicating that IHL is not directly related to fasting GCGN or AA levels in people with T2DM. By contrast, the reduction of liver fat resulted in a significant improvement of insulin sensitivity as shown by either HOMA-IR, Matsuda index, or *M*-value. Moreover, other markers of metabolism improved such as uric acid, CRP, and blood lipids (19, 20). Therefore, the HPD induced *meal related* increase most likely explains the metabolic improvements while fasting GCGN may be of minor importance.

The decrease in liver fat with HPDs was remarkably variable which might be related to hepatic GCGN resistance, because GCGN most likely drives the liver fat reduction by specifically enhancing hepatic lipolysis and inhibiting lipogenesis (24, 32). We therefore compared subjects above the median and below the median of liver fat reduction. The upper 50th percentile lost 27% of IHL which resulted in 12.7% IHL after the intervention while the lower 50th percentile lost 65% of liver fat which led to 4.6% IHL on average which is below the threshold definition of fatty liver. Although all indices of insulin resistance improved significantly only in the greater IHL-reduction group, there was no significant difference in fasting GCGN, GCGN–alanine index, or other fasting GCGN–AA indices. There were also no significant changes in the fasting levels of AA. This shows that changes of insulin sensitivity and GCGN sensitivity as calculated by the GCGN–alanine index in response to metabolic improvements can be dissociated in T2DM mellitus. Therefore, the alpha-cell response in the fasting state appears to be less responsive to reductions of liver fat than other metabolic parameters.

### Improvements of Meal-Related Insulin and Glucagon Responses

Meal related responses of GCGN are thought to be exaggerated in T2DM although this has received little attention with regards to responses to protein intake previously. We assessed whether the reduction of IHL would alter the GCGN response to protein intake. The same protein rich MMTTs were performed before and after the intervention such that each individual could serve as its own control. This showed a reduction of insulin and GCGN responses in the MMTTs in the presence of greater reduction of liver fat. Moreover, the levels of GCGN relative to insulin increased supporting a contribution of GCGN to the metabolic improvements.

### Altered Alanine Responses May Reflect Changes of the Glucose-Alanine Cycle

Remarkably, the plasma levels of alanine in the meal challenge tests were selectively reduced after 6 weeks of HPD, accompanied by a pronounced reduction of IHL, while the other AA and total AA did not change. The glucose-alanine cycle is well known to play a key role in glucose and AA metabolism (33). Alanine is generated by transamination from other AA used as energy substrates in muscle and transports amino groups to the liver which detoxifies the ammonium groups by delivery to the urea cycle. GCGN was shown to preferentially increase hepatic alanine uptake several-fold as compared to other AA (33). Alanine was recently shown to directly regulate mitochondrial oxidative metabolism in fasted humans (34). Mouse studies identified alanine as an intracellular activator of AMPK in hepatocytes which was dependent on ALT1 and the extraction of intermediate metabolites of the TCA-cycle (35). Alanine supplementation resulted in improved glucose metabolism of lean or obese mice. The alanine metabolic pathway was shown to be reversibly dysregulated in obese mice and humans and associated with impairments of ureagenesis (36, 37). We interpret the selective reduction of alanine in the MMTTs therefore as an indication of more effective use of alanine and improved mitochondrial oxidative function which may partially explain the improvements of glucose metabolism. As GCGN primarily regulates hepatic alanine uptake and metabolism, the reduced levels may indicate an improved prandial hepatic GCGN sensitivity. Notably, insulin resistance of protein metabolism was shown to be more pronounced in the fasting state while postprandial responses were close to normal (38). In analogy, postprandial GCGN actions may adapt preferentially to metabolic improvements.

A remarkable observation was that levels of urea were higher in subjects with greater liver fat reduction and showed a greater increase during the HPD intervention. This may indicate that there was a higher efficiency of GCGN to induce AA degradation and ureagenesis which may support the loss of IHL (32) as discussed above. In addition, GCGN was shown to specifically induce hepatic secretion of cAMP into the bloodstream to regulate kidney function which is a further energy-expensive signaling pathway (39). The improvements of IHL and insulin sensitivity in the entire cohort indicate a sufficiently preserved capacity of the liver to handle AA metabolism and to profit from its consequences in response to HPD. However, there appear to be subgroup-specific differences in the capacity to respond to HPD which are not well understood at present.

An important concern regarding high protein intake is a potential impairment of renal function due to the increased delivery of urea (40). GCGN was shown to participate in the adaptation of the kidney to increased protein intake (41). However, there is no conclusive evidence that limitation of protein intake prevents the progression of renal failure in T2D in randomized prospective studies (42, 43). Nevertheless, high protein intake should be avoided in patients with renal impairment until better evidence is available.

Limitations of the study apply to the relatively small number of patients who displayed a well-controlled non-insulin requiring diabetes and were characterized in considerable detail. The study used plant or animal protein supplements which differed in AA composition and there was a gender dysbalance in the groups above or below the median of liver fat reduction. The patients were Caucasian and of moderately advanced age. We did not study direct responses to exogenous administration of GCGN which may allow more sensitive assessment of GCGN responses. However, the high protein MMTTs reflect the real-life situation.

## Conclusion

Although fasting levels of GCGN are positively correlated with insulin resistance and IHL, increasing prandial GCGN secretion by HPD improves IHL, insulin sensitivity, fasting glucose, and circulating free saturated fatty acids. This associates with a selective reduction of alanine in meal challenge tests which is known to be primarily regulated by GCGN. Alanine links GCGN-stimulated glucose and AA-metabolism and might play a key role in augmenting insulin sensitivity and in inhibition of lipogenesis through AMPK-dependent pathways. Moreover, the metabolic improvements are associated with a reduction of meal stimulated insulin and GCGN secretion but a greater GCGN relative to insulin secretion. Together these findings suggest a primary role of prandial GCGN in the HPD-induced metabolic improvements which appears to be associated with an increased GCGN sensitivity.

## Data Availability Statement

The original contributions presented in the study are included in the article/Supplementary Material, further inquiries can be directed to the corresponding authors.

## Ethics Statement

The studies involving human participants were reviewed and approved by the Ethics Committee of the University of Potsdam. The patients/participants provided their written informed consent to participate in this study.

## Author Contributions

MM, OP-R, and SH conducted the experiments by dietary consultation and collected the data. SK, MM, OP-R, and SH analyzed and interpreted the experimental data. SR analyzed the samples, designed the study, and interpreted the data. JZ performed the statistical analysis, designed the figures and tables, and wrote the manuscript. AP designed the study and wrote the manuscript. All authors read and revised the manuscript, contributed to discussion, and approved the final version of this manuscript.

## Conflict of Interest

The authors declare that the research was conducted in the absence of any commercial or financial relationships that could be construed as a potential conflict of interest.

## Publisher’s Note

All claims expressed in this article are solely those of the authors and do not necessarily represent those of their affiliated organizations, or those of the publisher, the editors and the reviewers. Any product that may be evaluated in this article, or claim that may be made by its manufacturer, is not guaranteed or endorsed by the publisher.
