# Supplementary material for: High Protein Diets Improve Liver Fat and Insulin Sensitivity by Prandial but Not Fasting Glucagon Secretion in Type 2 Diabetes
Source: Front Nutr. 2022 May 19;9:808346. doi: 10.3389/fnut.2022.808346 (PMC9160603; doi:10.3389/fnut.2022.808346)
Supplement: Supplementary file 1 [file Data_Sheet_1.PDF]

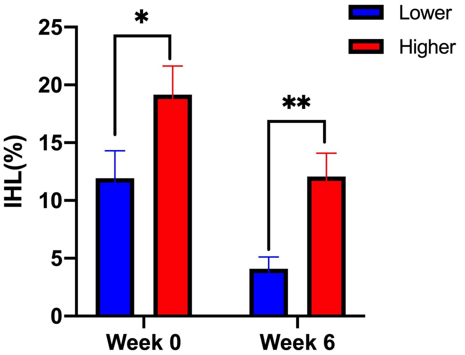

Figure S1: : IHL(%) change in lower (below median, blue) and higher (above median, red) reduction of intrahepatic lipid content (IHL) .*p<0.05; **p<0.01.

	Lower liver fat reduction (n = 16)	Higher liver fat reduction (n =15)	p value
Gender (male/female)	8m/8f	11m/4f	/
PP/AP	9/7	7/8	/
Protein intake (%EN)	16.8±3.4	17.4±2.4	0.57
Total protein intake (g)	83.1±23.0	90.2±22.3	0.39

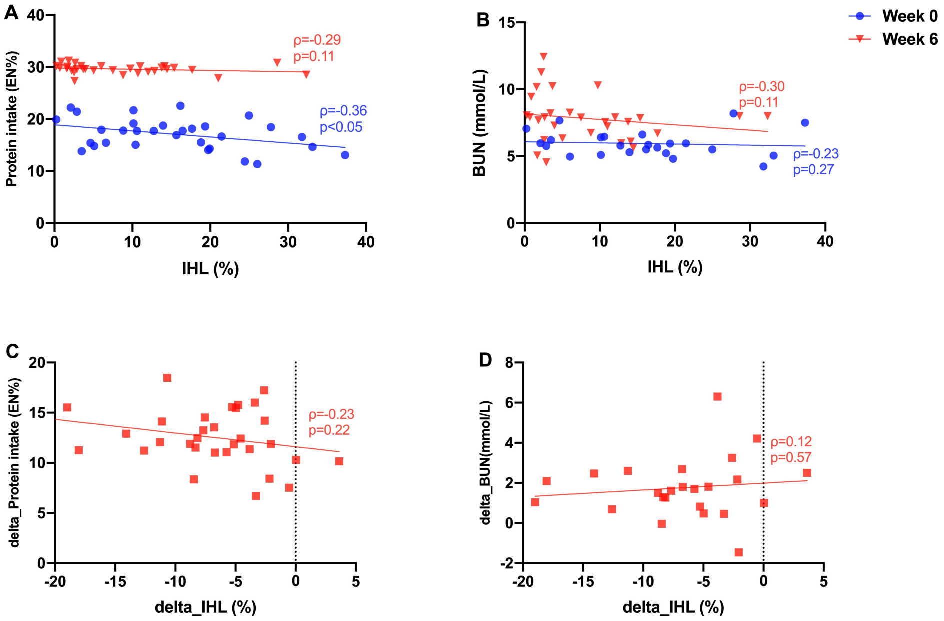

Figure S2: Protein intake at baseline (blue)and after 6 weeks (red) calculated from 3 day food protocols and calculated with the Computer program Prodi. The data show that the baseline protein intake varied but was very homogenous at the end of the study. The change in protein intake (C) and blood urea nitrogen (D) before the intervention to that during the intervention did not correlate with the loss of liver fat. *p<0.05; **p<0.01.

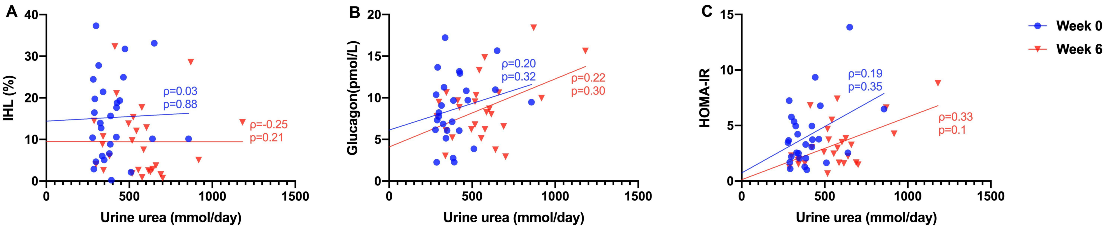

Figure S3: Correlations between 24h-urine urea and (A) IHL (%); (B) glucagon; (C) HOMA-IR before (Week 0, blue) and after the intervention (Week 6, red). *p<0.05; **p<0.01.

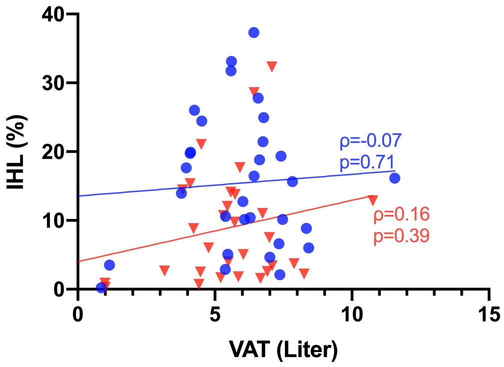

Figure S4: Correlations between VAT and IHL (%) before (Week 0, blue) and after the intervention (Week 6, red). There is no correlation between VAT and liver fat at baseline and after intervetion.

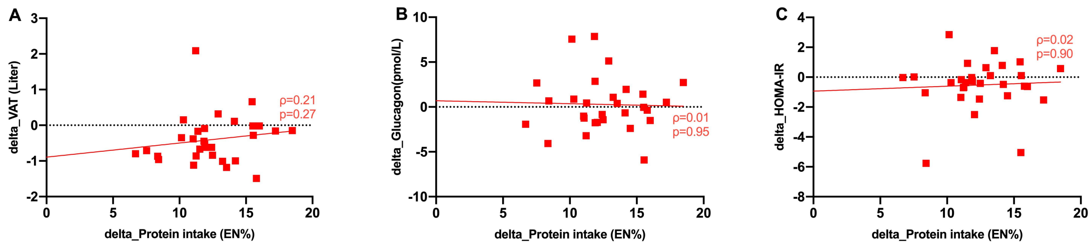

Figure S5: Correlations between the change of protein intake (EN%) and (A) the change of VAT; (B) the change of glucagon; (C) the change of HOMA-IR after 6-week intervention.
