# Supplementary material for: High Protein Diets Improve Liver Fat and Insulin Sensitivity by Prandial but Not Fasting Glucagon Secretion in Type 2 Diabetes
Source: Front Nutr. 2022 May 19;9:808346. doi: 10.3389/fnut.2022.808346 (PMC9160603; doi:10.3389/fnut.2022.808346)
Supplement: Supplementary file 2 [file Data_Sheet_2.docx]

**Supplementary tables**

**Table S1 Amino-acid levels in plasma and GCGN-amino acids indices (n=31) at baseline (Week 0) and after HPD intervention (Week 6)**

| Parameters (n=31) | Week 0 | Week 6 | *p* |
| --- | --- | --- | --- |
| Total AA (μmol/L) | 2655.2±577.3 | 2607.3±422.2 | 0.52 |
| BCAA (μmol/L) | 349.2±85.5 | 342.6±60.2 | 0.63 |
| Alanine (μmol/L) | 200.2±64.3 | 181.5±42.1 | 0.16 |
| Glutamine (μmol/L) | 928.9±233.6 | 887.0±185.1 | 0.32 |
| Arginine (μmol/L) | 51.5±15.5 | 48.6±10.9 | 0.17 |
| Serine (μmol/L) | 50.9±22.1 | 54.4±16.9 | 0.20 |
| Phenylalanine (μmol/L) | 44.0±9.3 | 43.8±9.0 | 0.92 |
| Asparagine (μmol/L) | 23.0±8.3 | 23.9±7.8 | 0.45 |
| Leucine (μmol/L) | 177.3±44.6 | 169.0±30.8 | 0.24 |
| Isoleucine (μmol/L) | 78.3±21.2 | 76.7±16.7 | 0.58 |
| Glucagon-total AA index | 22.1±11.3 | 21.7±10.9 | 0.77 |
| Glucagon-BCAA index | 2.9±1.5 | 3.0±1.7 | 0.80 |
| Glucagon-Alanine index | 1.7±1.0 | 1.5±0.74 | 0.86 |
| Glucagon-Glutamine index | 7.6±3.7 | 7.5±3.9 | 0.89 |
| Glucagon-Arginine index | 0.42±0.23 | 0.4±0.23 | 0.69 |
| Glucagon-serine index | 0.41±0.22 | 0.44±0.21 | 0.30 |
| Glucagon-Phenylalanine index | 0.36±0.18 | 0.37±0.2 | 0.82 |
| Glucagon-Asparagine index | 0.19±0.12 | 0.2±0.11 | 0.21 |
| Glucagon-Leucine index | 1.48±0.78 | 1.46±0.89 | 0.99 |
| Glucagon-Isoleucine index | 0.64±0.33 | 0.65±0.37 | 0.80 |

AA: amino acid; BCAA: branch-chained amino acid.

**Table S2 Levels of amino-acids and GCGN-AA index at baseline (week 0) and after HPD intervention (week 6) with lower (below median) and higher (above median) reduction of intrahepatic lipid content (IHL)**

| **Parameters (n=31)** | **Lower liver fat reduction, n = 16** | | | **Higher liver fat reduction, n =15** | | | ***p***Week6-Week0 |
| --- | --- | --- | --- | --- | --- | --- | --- |
|  | **Week 0** | **Week 6** | ***p*** | **Week 0** | **Week 6** | ***p*** |  |
| Total AA | 2839.8±480.1 | 2756.5±306.5 | NS | 2444.2±622.5 | 2458.1±477.2 | NS | NS |
| BCAA | 377.1±73.1 | 359.0±47.1 | NS | 319.5±90.0 | 325.2±69.0 | NS | NS |
| Alanine | 212.2±56.2 | 201.6±42.2 | NS | 187.4±71.7 | 161.5±32.0 | NS | NS |
| Glutamine | 990.9±200.6 | 949.3±186.0 | NS | 862.7±254.3 | 820.5±164.8 | NS | NS |
| Arginine | 53.1±15.3 | 49.0±11.2 | NS | 49.8±16.2 | 48.2±11.0 | NS | NS |
| Serine(μmol/L) | 57.8±23.6 | 60.5±16.7 | NS | 43.5±18.4 | 47.9±15.1 | NS | NS |
| Phenylalanine(μmol/L) | 47.0±8.7 | 45.8±8.6 | NS | 40.7±9.0 | 41.6±9.2 | NS | NS |
| Asparagine(μmol/L) | 25.8±6.6 | 25.2±8.4 | NS | 19.7±9.1 | 22.6±7.0 | NS | NS |
| Leucine(μmol/L) | 191.2±37.9 | 178.0±26.7 | NS | 162.5±47.6 | 159.5±32.8 | NS | NS |
| Isoleucine(μmol/L) | 85.6±19.9 | 80.5±15.0 | NS | 70.5±20.2 | 73.0±18.0 | NS | NS |
| Glucagon-Alanine index | 1.7±0.8 | 1.8±0.8 | NS | 1.7±1.3 | 1.2±0.6 | NS | NS |
| Glucagon-Glutamine index | 7.9±3.2 | 8.9±4.3 | NS | 7.2±4.2 | 6.0±2.8 | NS | NS |
| Glucagon-Arginine index | 0.42±0.21 | 0.45±0.28 | NS | 0.42±0.25 | 0.35±0.16 | NS | NS |
| Glucagon-total AA index | 22.7±9.1 | 25.4±12.0 | NS | 21.4±13.6 | 16.8±9.4 | NS | NS |
| Glucagon-BCAA index | 3.0±1.2 | 3.4±1.9 | NS | 2.8±1.8 | 2.5±1.4 | NS | NS |
| Glucagon-Serine index | 0.45±0.2 | 0.53±0.22 | NS | 0.38±0.24 | 0.34±0.16 | NS | NS |
| Glucagon-Phenylalanine index | 0.38±0.16 | 0.42±0.21 | NS | 0.35±0.21 | 0.32±0.19 | NS | NS |
| Glucagon-Asparagine index | 0.21±0.11 | 0.23±0.12 | NS | 0.17±0.13 | 0.17±0.11 | NS | NS |
| Glucagon-Leucine index | 1.51±0.62 | 1.69±1.01 | NS | 1.46±0.94 | 1.2±0.67 | NS | NS |
| Glucagon-Isoleucine index | 0.67±0.28 | 0.75±0.42 | NS | 0.61±0.38 | 0.54±0.29 | NS | NS |

AA: amino acid; BCAA: branch-chained amino acid. NS, not significant (P>0.05).

**Table S3 Correlations between IHL and other parameters at baseline (Week 0) and after HPD intervention (Week 6) calculated for the whole study group.**

| **Parameters (n=31)** | **Week 0** | **Week 6** |
| --- | --- | --- |
| HOMA-IR | ρ=0.554  *p*<0.01** | ρ=0.592  *p*<0.001*** |
| Glucagon | ρ=0.454  *p*=0.012* | ρ=0.552  *p*=0.002** |
| Glucagon-total AA index | ρ=0.329  *p*=0.081 | ρ=0.554  *p*=0.002** |
| Glucagon-BCAA index | ρ=0.377  *p*=0.04* | ρ=0.645  *p*<0.001*** |
| Glucagon-Alanine index | ρ=0.369  *p*=0.045* | ρ=0.652  *p*<0.001*** |
| Glucagon-Glutamine index | ρ=0.208  *p*=0.27 | ρ=0.622  *p*<0.001*** |
| Glucagon-Arginine index | ρ=0.274  *p*=0.142 | ρ=0.527  *p*=0.003** |
| Glucagon-Histidine index | ρ=0.386  *p*=0.035* | ρ=0.586  *p*<0.01** |
| Glucagon-Serine index | ρ=0.163  *p*=0.39 | ρ=0.571  *p*<0.01** |
| Glucagon-Phenylalanine index | ρ=0.357  *p*=0.053 | ρ=0.594  *p*<0.01** |
| Glucagon-Asparagine index | ρ=0.29  *p*=0.127 | ρ=0.464  *p*=0.011* |
| Glucagon-Leucine index | ρ=0.376  p=0.041* | ρ=0.593  *p*=0.001** |
| Glucagon-Isoleucine index | ρ=0.392  *p*=0.032* | ρ=0.64  *p*<0.001*** |

HOMA-IR: homeostatic model assessment for insulin resistance; AA: amino acid; BCAA: branch-chained amino acid.

**p*<0.05; ***p*<0.01; ****p*<0.001.

**Table S4 Insulin/GCGN ratio in MMTT1 at baseline (Week 0) and after HPD intervention (Week 6) compared between lower (below median) and higher (above median) of intrahepatic lipid (IHL) reduction groups**

| **Parameter** | **Lower liver fat reduction (n = 16)** | | | **Higher liver fat reduction (n =15)** | | | ***p***Week6-Week0 |
| --- | --- | --- | --- | --- | --- | --- | --- |
|  | **Week 0** | **Week 6** | ***p*** | **Week 0** | **Week 6** | ***p*** |  |
| Insulin/glucagon ratio(Fasting) | 1.0±0.49 | 0.87±0.44 | 0.069 | 1.25±0.94 | 0.93±0.42 | 0.3 | 0.91 |
| Insulin/ glucagon ratio(60min) | 3.4±1.8 | 3.5±2.2 | 0.47 | 5.4±1.2 | 4.2±2.1 | 0.28 | 0.20 |
| Insulin/ glucagon ratio(120min) | 3.9±2.4 | 3.7±2.3 | 0.93 | 4.5±2.9 | 4.5±2.1 | 0.73 | 0.85 |
| Insulin/ glucagon ratio(180min) | 2.6±1.6 | 2.5±1.6 | 0.72 | 2.4±1.6 | 2.4±0.82 | 0.18 | 0.28 |

**Table S5 Quantification of amino acids at baseline (Week 0) and after HPD intervention (Week 6) during the mixed meal tolerance tests MMTT1 and MMTT2 (AUC *360min) in the entire study group.**

| **Parameter(n=31)** | **Week 0** | **Week 6** | ***p* value** |
| --- | --- | --- | --- |
| AUC Alanine (μmol/L) | 76102.0±12995.2 | 65682.2±12045.7 | <0.001*** |
| AUC Taurine (μmol/L) | 13229.2±4022.7 | 10747.1±2527.2 | <0.05* |
| AUC Glutamine (μmol/L) | 593380.4 ±159241.8 | 582119.7±136966.5 | 0.63 |
| AUC Arginine (μmol/L) | 18067.3±5544.7 | 18603.7±5733.9 | 0.43 |
| AUC Valine (μmol/L) | 44089.0±10210.2 | 45903.6±11797.7 | 0.39 |
| AUC Methionine (μmol/L) | 9682.8±3472.6 | 9412.0±3545.5 | 0.32 |
| AUC Lysine (μmol/L) | 374134.4±100709.6 | 360344.2±76751.5 | 0.41 |
| AUC Tryptophane (μmol/L) | 3393.3±830.4 | 3266.1±1044.0 | 0.47 |
| AUC Tyrosine (μmol/L) | 19187.7±5253.0 | 17768.4±4869.6 | 0.20 |
| AUC Histidine (μmol/L) | 17122.6±4006.9 | 15703.4±4180.6 | 0.16 |
| AUC Serine (μmol/L) | 22257.0±7107.5 | 21667.3±6015.4 | 0.66 |
| AUC Phenylalanine (μmol/L) | 27640.8±8889.4 | 27510.2±8311.1 | 0.88 |
| AUC Asparagine (μmol/L) | 9900.9±2438.2 | 10520.4±4111.5 | 0.39 |
| AUC Leucine (μmol/L) | 88800.6±16271.8 | 89733.3±15626.9 | 0.70 |
| AUC Isoleucine (μmol/L) | 26189.8±5058.5 | 26539.6±5931.0 | 0.68 |

AUC: area under curve.**p*<0.05; ****p*<0.001.

**Table S6 Quantification of amino acids at baseline (Week 0) and after HPD intervention (Week 6) during the mixed meal tolerance tests MMTT1 and MMTT2 AUC (*360min) in the groups split by lower (below median) and higher (above median) IHL reduction groups.**

| **Parameters** | **Lower liver fat reduction (n = 16)** | | | **Higher liver fat reduction (n =15)** | | |
| --- | --- | --- | --- | --- | --- | --- |
|  | **Week 0** | **Week 6** | ***p*** | **Week 0** | **Week 6** | ***p*** |
| AUC Alanine | 78331.6±16265.1 | 67073.3±14093.3 | <0.05* | 74350.2 ±10037.6 | 64589.2 ±10590.8 | <0.01** |
| AUC Taurine | 13269.1±3153.8 | 11012.5±2511.5 | 0.074 | 13197.9 ±4714.0 | 10538.7 ±2614.0 | 0.067 |
| AUC Glutamine | 622437.6±176752.7 | 573662.1±131730.1 | 0.26 | 569166.1 ±146431.6 | 589167.7 ±146619.4 | 0.40 |
| AUC Arginine | 18057.9±5817.5 | 19307.3±6112.4 | 0.17 | 18075.3 ±5542.8 | 18008.4 ±5571.6 | 0.95 |
| AUC Valine | 46787.5±11094.0 | 47830.0±13369.2 | 0.77 | 41598.1 ±9037.3 | 44125.4 ±10363.1 | 0.33 |
| AUC Methionine | 9216.5±3874.4 | 8725.1±3469.1 | 0.098 | 10077.3 ±3199.4 | 9993.3 ±3642.2 | 0.85 |
| AUC Lysine | 426551.3±105244.8 | 400641.0±77631.6 | 0.40 | 326482.7 ±71209.1 | 323710.9 ±57075.0 | 0.88 |
| AUC Tryptophane | 3286.3±988.3 | 3087.6±1208.5 | 0.43 | 3483.9 ±698.8 | 3417.2 ±904.5 | 0.80 |
| AUC Tyrosine | 19334.6±6115.1 | 16830.4±3287.5 | 0.11 | 19053.2 ±4599.2 | 18628.3 ±5992.9 | 0.79 |
| AUC Histidine | 18163.3±4293.0 | 15485.8±4541.9 | 0.15 | 16242.1 ±3685.3 | 15887.5 ±4028.3 | 0.75 |
| AUC Serine | 22112.4±7349.1 | 21113.3±5906.1 | 0.70 | 22377.5 ±7226.0 | 22128.9 ±6327.0 | 0.85 |
| AUC Phenylalanine | 28380.3±7830.1 | 27727.1±6329.7 | 0.38 | 26958.2 ±10039.4 | 27310.0 ±10066.3 | 0.83 |
| AUC Asparagine | 10917.3±2827.7 | 10831.9±4460.2 | 0.95 | 9053.9 ±1756.3 | 10260.7 ±3979.3 | 0.18 |
| AUC Leucine | 90773.3±13975.0 | 93413.1±14884.6 | 0.49 | 87131.4 ±18388.7 | 86619.6 ±16140.3 | 0.88 |
| AUC Isoleucine | 27598.2±4480.7 | 28970.9±6540.0 | 0.27 | 24998.1 ±5381.2 | 24482.3 ±4666.5 | 0.66 |

AUC: area under curve. **p*<0.05; ***p*<0.01.

**Table S7** **Correlations between GCGN and levels of FFA before (Week 0) and after (Week 6) HPD intervention in the entire study group**

| **Parameter (n=31)** | **Week 0** | **Week 6** |
| --- | --- | --- |
| C16:1n7c | ρ=0.303  *p*=0.11 | ρ=0.417  *p*<0.05* |
| C18:3n6 | ρ=0.229  *p*=0.232 | ρ=0.417  *p*<0.05* |
| C22:5n6 | ρ=-0.426  *p*<0.05* | ρ=-0.004  *p*=0.982 |
| C18.1n9c | ρ=0.359  *p*=0.056 | ρ=0.265  *p*=0.165 |
| C18.1n7c | ρ=0.263  *p*=0.169 | ρ=0.171  *p*=0.375 |
| C18.2n6c | ρ=-0.028  *p*=0.885 | ρ=0.034  *p*=0.862 |
| C20.3n6 | ρ=0.119  *p*=0.540 | ρ=0.355  *p*=0.059 |
| C20.4n6 | ρ=0.346  *p*=0.066 | ρ=0.116  *p*=0.548 |
| C22.5n3 | ρ=0.063  *p*=0.747 | ρ=0.269  *p*=0.158 |
| C22.6n3 | ρ=0.027  *p*=0.891 | ρ=0.155  *p*=0.421 |
| elongase activity index(C18.0/C16.0) | ρ=-0.126  *p*=0.516 | ρ=-0.174  *p*=0.366 |
| ∆6 desaturase activity index (C18:3n6/C18:2n6) | ρ=0.302  *p*=0.111 | ρ=0.303  *p*=0.11 |
| ∆5 desaturase activity index (C20:4n6/C20:3n6) | ρ=-0.015  *p*=0.937 | ρ=-0.381  *p*<0.05* |

*p<0.05.
