# Supplementary material for: High Protein Diets Improve Liver Fat and Insulin Sensitivity by Prandial but Not Fasting Glucagon Secretion in Type 2 Diabetes
Source: Front Nutr. 2022 May 19;9:808346. doi: 10.3389/fnut.2022.808346 (PMC9160603; doi:10.3389/fnut.2022.808346)
Supplement: Supplementary file 3 [file Data_Sheet_3.doc]

**
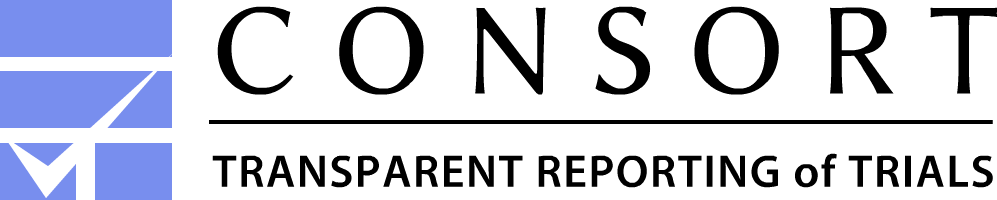
**

**CONSORT 2010 Flow Diagram**

**Allocation**

**Analysis**

**Follow-Up**

**Enrollment**

Assessed for eligibility (n=85)

Excluded (n=41)

♦  Not meeting inclusion criteria (n=22)

♦  Declined to participate (n=16)

♦  Other reasons (n=3)

Analysed (n=15)
♦ Excluded from analysis (No 1H-MRS measurement of the liver before and after intervention) (n=3)

Discontinued intervention (medical reason n=2; lost interest n=2)

Allocated to intervention (n=22)

♦ Received allocated intervention (high animal protein) (n=22)

♦ Did not receive allocated intervention (n=0)

Discontinued intervention (lost interest n=2; obtained no vein access n=1)

Allocated to intervention (n=22)

♦ Received allocated intervention (high plant protein diet ) (n=22)

♦ Did not receive allocated intervention (n=0)

Analysed (n=16)
♦ Excluded from analysis (No 1H-MRS measurement the liver before and after intervention) (n=3)

Randomized (n=44)
